# Acquisition of CD4-Dependence by CD4-Independent SIV Passaged in Human Peripheral Blood Mononuclear Cells

**DOI:** 10.1186/1742-4690-9-61

**Published:** 2012-07-25

**Authors:** Sujatha Iyengar, David H Schwartz

**Affiliations:** 1 Department of Molecular Microbiology and Immunology, Bloomberg School of Public Health, Johns Hopkins University, Baltimore, USA; 2 Current address: Jurist Research Department, Hackensack University Medical Center, 40 Prospect Avenue, Hackensack, NJ, 07601, USA

**Keywords:** SIV, Tropism, CD4 independent, Chemokine receptor, Selection, Evolution

## Abstract

**Background:**

Chemokine receptors (CKRs), the primordial receptors for primate lentiviruses, are sufficient to mediate virus-cell fusion. Several different fusogenic CKRs and related receptors provide a broad potential host cell range, presumably advantageous for viral spread within a given infected individual, and across species. By contrast, the additional constraint of obligatory CD4 binding, just prior to CKR engagement, radically restricts potential host cells within an individual (or lymph node microenvironment), and might also limit xenotransmission, as CD4 sequences vary among primates. In spite of these potential drawbacks, CD4 dependent entry for SIV and HIV is the rule rather than the exception, and is generally thought to have evolved by selection for 1) stabilization of virus–cell surface interactions, and 2) conformational shielding of readily neutralized CKR binding epitopes. CD4 binding residues of SIV and HIV envelope are recessed, (relatively hidden from immune detection) and may exhibit a strong degree of automimicry, thus benefitting from self tolerance.

Documented evolution, within individual macaques, of neutralization-resistant CD4-dependent SIV, derived from CD4-independent inocula, supports these ideas, but does not explain CD4’s exclusive role as the penultimate receptor-even more striking, given the wide diversity of CKRs and other surface molecules that can serve as actual fusion receptors for SIV. We, therefore, explored the additional, non-exclusive, hypothesis that surface CD4 on leukocytes is a marker of a more favorable host cell environment, as compared to CD8, NK, or B cell surface markers.

**Results:**

We demonstrate progressive in vitro evolution of two SIV strains to CD4-dependence (and CXCR4 tropism) in normal human PBMCs (hPBMCs). The two CD4-independent strains of SIV tested developed nearly complete CD4 dependence over several months of serial passage in hPBMCs, correlating with a limited number of non-synonymous *env* region mutations, some previously reported to be determinants of CD4-dependency. The initial ability of SIV stocks to grow to significant (albeit, relatively low) levels in CD4(−), CD14(−) cells was also lost with long term passage. Rapid emergence and subsequent prominence of G → A and A → G mutations within *env* regions associated with CD4 dependence was seen.

**Conclusions:**

Progressive acquisition of strict CD4 tropism, independent of immunoselection, supports the idea that surface CD4 identifies optimal host cells having intracellular environments most favorable to viral replication. The prominence of mutations involving G to A, or A to G, suggests that APOBEC 3 mediated infidelity may facilitate rapid switching of cell surface receptor usage within SIV swarms encountering fluctuating availability of optimal CD4^+^CKR^+^ targets. These observations of non-immune selection are compatible with, and may accelerate, simultaneous selection for previously described CD4-dependent neutralization resistance in vivo.

## Background

SIV has crossed many primate species barriers during its evolution to HIV [1,2], and the widely divergent outcomes of infection (from benign subclinical to rapidly pathogenic) reflect multiple host – virus interactions. Among these are coreceptor usage [3,4], and cell subset tropism within progressively colonized microanatomic niches [5-7]. Lin et al. [8] have summarized the evidence that chemokine receptors (CKRs) were the primordial lentivirus receptors, with the evolution of penultimate CD4 binding a more recent development in primate hosts. Most SIV (and essentially all HIV) exhibit strong CD4 dependence, which is generally thought to reflect the selective advantage of high affinity CD4 binding for stabilization of cell-virion interactions, and shielding of chemokine receptor (CKR) binding sites from neutralizing antibodies [9,10].

Yet, with respect to stabilizing membrane interactions, other cognate pairings (e.g., viral membrane ICAM – host membrane LFA-1) stabilize cell-virus coupling during retrovirus docking. Similarly, the explanation of CD4 dependence as a means of protecting readily neutralized fusogenic CKR binding epitopes begs the question: “*Why, despite fusogenic receptor diversity for SIV and HIV, has CD4 emerged as the exclusive selecting co-receptor, rather than functionally analogous CD8, or some ubiquitous surface molecule (*e.g.*, CD45)?”* Our earlier demonstration that CD4 co-caps with CKRs on the surface of gp120 or virion exposed huPBMCs, and that blocking this capping prevents infection [11] does not, by itself, explain why CD4 should have eclipsed all other molecules in this role, unless it were unique among surface molecules in its actin-mediated association with CKRs. This would appear to be ruled out by work of Tardif and Tremblay [12,13] demonstrating actin-mediated LFA-1 clustering, post-HIV binding, and by observations from several laboratories (e.g., ref [14]) on the co-clustering of LFA-1, CD8, and CKRs at the immunological synapse of CTLs.

CD4-independent SIV and HIV-2 strains can be selected in vitro [1,8,9,15,16], and isolated from the CNS of macaques [17,18] and blood of rapid progressor (RP) or late stage [10,19] animals – settings with a paucity of CD4+ targets. Similarly, CD4 independent HIV-1 has been recovered from the CNS of HIV + individuals and (very rarely) AIDS patients with extreme CD4+ cell depletion, in some cases from CD8+ cells [20,21]. Of note, Vodros et al., [3] demonstrated, within 12 days of infection, increased heterogeneity of circulating SIV envelopes capable of mediating CD4-independent fusion in vitro. They suggested that local depletion of CD4+ T cells available, within initially infected sites during the acute phase, might select for CD4 independence.

Replacement of injected CD4-independent RP SIV by CD4-dependent virus in macaques has been attributed to neutralizing antibodies (Abs) arising roughly 40 weeks post infection, while (rare) persistence of CD4-independent SIV in RP animals was interpreted as the consequence of feeble, ineffective, neutralizing Ab responses [10,19,22]. But RP macaques also have rapid loss of almost all susceptible CD4+ lymphocytes, so, likewise, non-immune selective pressures favoring CD4 tropism would be absent, allowing persistent CD4-independence at the level of non-immune selection, as well. Decreased fitness in macaque PBMCs of CD4 independent SIV from rapid progressors (vs. parental CD4-dependent virus) was considered in vitro artifact [10,22]. However, rapid progressor (RP) SIV was not examined for in vitro evolution to parental CD4 dependent phenotype and/or genotype during maintenance in vitro. Referring to data not shown, Vodros et al.[3] noted that “extensive passage on hPBMCs” of a largely CD4-independent SIV strain “resulted in inoculum from which strictly CD4-dependent clones were derived, suggesting that CD4-dependent viruses may be more fit under these conditions.”

One previously unexplored potential pressure for the selection of CD4 as the exclusive co-receptor for HIV is that CD4 cells are intrinsically better internal hosts for SIV (and HIV) replication than other primary cells, for reasons (e.g., cell-type specific restriction factors) unrelated to surface CD4. This hypothesis predicts that, among a swarm of SIV, CD4 dependent phenotypes will out-compete strains with different (e.g. CD8 co-receptor) or more promiscuous (e.g., CKR only) entry criteria, because they selectively infect host cells that are intrinsically more productive. This advantage should exist in the absence of specific anti-SIV humoral or cell mediated immunity, as long as there are sizable proportions of both CD4+ and CD4(−) targets with suitable surface CKRs for binding and fusion. Selection for CD4 dependence might diminish in the face of drastically reduced availability of activated CD4+ cells (as in RP macaques, or macaque CNS infection, or within a “burned out” node, depleted of in situ CD4+ activated T cells), or during xenotransmission to a primate whose CD4 had much lower avidity for envelope.

We set out to test the scenario of selection for CD4 tropism in the absence of immune pressure, by long term culturing in normal hPBMCs of two SIV strains which exhibit CD4 independence during initial rounds of infection in hPBMCs [23,24].

## Results

### SIV stock strains are CD4 independent in hPBMCs, and productively infect hPBMCs depleted of CD4+ and CD14+ cells

SIV mac239 _CEMx174_ and 17E-Fr stocks, both produced in CEMx174 cultures, were inoculated into wells containing PHA stimulated hPBMCs. After continuous passage of SIV mac239 in CEM x 174 cells, the resulting stock, which we here call SIV mac239 _CEMx174_, is CD4 independent for infection of human PBMCs, while remaining CD4 dependent in macaque PBMCs, as previously reported [23,24]. This fortuitous observation gave us a second SIV strain, along with SIV 17E-Fr (which was genetically unchanged after passage in CEMx174 cells) to analyze for progressive acquisition of CD4 dependence in hPBMCs. This phenotype was not due to surface components incorporated from the CEMx174 line, in which case CD4 independence should have been lost after just one round of replication in hPBMCs. Rather, sequencing of stock SIV mac239 _CEMx174_ indicates the presence of several mutations differing from the parental SIV mac239 [GenBank accession number M33262], and previously associated with CD4 independence (discussed below).

**Figure 1:**
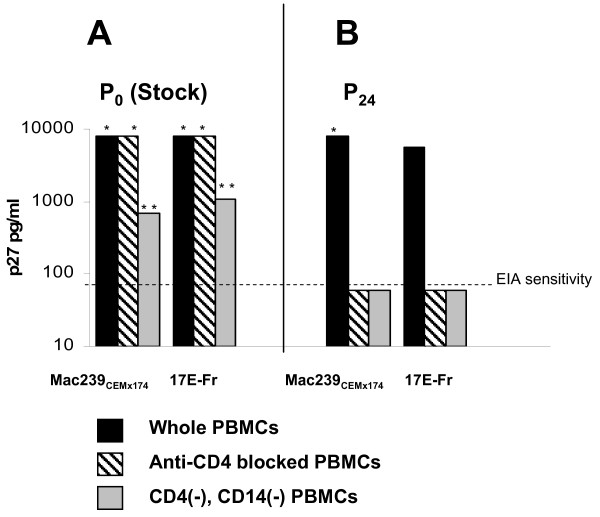
**Stock vs. Passage 24 SIV CD4 dependence and infection of CD4+ and CD14+ depleted hPBMCs. A.** Day 7 growth of stock SIV mac239 _CEMx174_ and SIV 17E-Fr in human PBMCs was robust (> 8,000 pg/ml p27) whether or not cells were pretreated with CD4 blocking concentrations of Leu3a. Limited supernatant volumes prevented endpoint dilutions, but contained p27 levels that remained off-scale (*) at 1:10 dilution. ImmunodepletedhPBMCs containing fewer than 1% CD4+ or CD14+ cells still supported readily detectable levels of viral replication, but at significantly lower levels (** p < 0.0001 vs. 8,000 pg/ml). **B.** By the 24^th^ passage in allogeneic hPBMCs, both SIV mac239_CEMx174_ and SIV 17E-Fr had lost the ability to infect PBMCs pretreated with anti-CD4. Moreover, no growth of either strain was detectable above EIA sensitivity (dashed line) in day 7 supernatants of hPBMC depleted of CD4+ and CD14+ cells.

**Figure 2:**
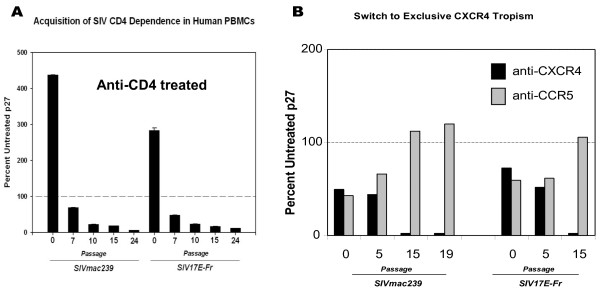
**Progressive changes in CD4 and CKR usage by SIV serially passaged in hPBMCs. A.** Day 7 supernatant p27 concentrations of hPBMC cultures, transiently treated with Leu3a anti-CD4 mAb during infection, were normalized to simultaneously mock-treated control cultures (100%, dashed line ------). Initial 4- and 3-fold ***greater*** replication in Leu3a anti-CD4 mAb treated cultures of, respectively, SIV mac239 _CEMx174_ (abbreviated SIV mac239) and SIV 17E-Fr was followed by progressively increasing CD4 dependence during serial passage in unrelated human donor PBMCs. **B**. By the 15^th^ passage, when both SIV strains had become almost completely CD4 dependent (see **A**), CKR usage in both strains had switched from roughly equal CXCR4 and CCR5 to nearly exclusive CXCR4 tropism, demonstrated by relative reduction of virus growth in cells pretreated with blocking mAbs against CXCR4 vs. CCR5.

**Figure 3:**
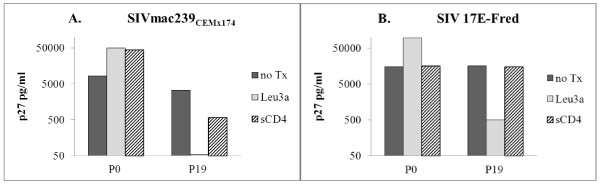
**Different effects of soluble CD4 on SIV mac239 CEMX174 vs. SIV 17E-Fred. A.** For SIV 239_CEMX174_, sCD4 (striped), like anti-CD4 (Leu3a, gray), enhanced infectivity of stock virus, but decreased growth of late passage virus, although to a lesser degree than Leu3a. **B**. For SIV 17E-Fred, sCD4 appeared to have no effect on stock or P19 virus infectivity. ***(Note that P0 p27 values reflect day 10 culture supernatants*vs.*day 7 for P19).***

Parallel cultures of equal numbers of anti-CD4 treated hPBMCs, or PBMCs immunodepleted of CD4+ and CD14+ cells, were established at the same time. Separate experiments demonstrated comparable levels of day 5 proliferation, as measured by thymidine incorporation, among these various cell populations (data not shown). As shown in Figure 1A, anti-CD4 mAb treated hPBMCs supported high replication (> 8,000 pg/ml p27) of both initial stock strains of SIV (off-scale at 1:10 dilution), as did untreated PBMCs. The Figure 1A supernatants were not diluted to endpoint, but in similar experiments using the stock viruses, anti-CD4 mAb treated cultures supported several fold higher levels of p27 production than untreated control cultures, as determined by endpoint dilution of day 7 or day 10 supernatants (Figures 2 and 3, discussed below).

Lack of surface CD4 dependence for entry does not formally prove that cells other than CD4+ lymphocytes are actually being infected. CD4+ cells might still be selectively targeted, via some non-CD4 mediated mechanism. This point was addressed by documenting moderate growth in hPBMCs rigorously depleted of CD4+ lymphocytes and CD14+ monocytes (< 1% CD4+, < 1% CD14+, as determined by flow cytometric analysis), albeit with significantly lower levels of virus production (~ 700 and 1100 pg/ml p27, respectively, of SIV mac239 _CEMx174_ and 17E-Fr, Figure 1A). The decreased growth of SIV in CD8+ T cells, B cells, and other remaining CD4(−) CD14(−) mononuclear cells, compared to anti-CD4 treated whole PBMCs, suggests that, despite blocking and/or downregulation of surface CD4, the CD4+ cells are still being infected, and are producing more virus than the CD4(−) cells from the same source. This is consistent with our hypothesis of a selective growth advantage for virions entering CD4+ lymphocytes when they are available, even if surface CD4 is not utilized for entry. This level of replication was absent in identically depleted cultures of late passage, CD4 dependent, SIV, which produced only uninfected control background levels of p27 (Figure 1B).

### CD4 independent SIV passaged repeatedly in hPBMCs becomes CD4 dependent in hPBMCs, exclusively CXCR4 tropic, and no longer infects hPBMCs depleted of CD4+ and CD14+ cells

The cultures established in hPBMCs (described above) were re-fed weekly with fresh allogeneic PHA stimulated normal hPBMCs and complete media. Table 1 shows that significant percentages of the non-adherent CD4(−), as well as CD4+ cells present in these cultures at days 1, 4, and 7 were positive for either CXCR4 or CCR5 (or both). Moreover, the increasing ratio of CXCR4 : CCR5 expression during the 7 day culture period was not markedly different for CD4+ vs. CD4(−) populations.

**Table 1:** Percentage of CD4+ and CD4(−) non-adherent hPBMCs expressing receptors for SIV during one week cultures

	**Percentage of Cells Expressing Surface Markers**			
**Surface markers:**	**CD4(−)CXCR4+**	**CD4(−)CCR5+**	**CD4 + CXCR4+**	**CD4 + CCR5+**
Day 1	37	22	47	8
Day 4	25	17	68	14
Day 7	53	7	36	4

Whole human PBMCs were stimulated with PHA + IL-2, and maintained in complete medium (10% FCS) for one week, with sampling of resuspended cells on days 1, 4, and 7. Cells were dual stained with CD4 and CXCR4 or CCR5 specific mAbs, to determine the percentages of CD4+ and CD4(−) cells expressing CXCR4 or CCR5 at various times. Because co-expression of CXCR4 and CCR5 on either CD4+ or CD4(−) cells was not determined, total percentages of CKR + cells are not calculated by summing the four populations for each day.

Aliquots of supernatant from SIV stocks (~10^3^ TCID_50_) or progressive weekly passages (P#) were sampled at 1–2 month intervals for sequencing, and used to infect normal PHA stimulated hPBMCs, with or without pre-blocking by anti-CD4 mAb (samples from P0, P7, P15, and P24), or anti-CKR mAb (samples from P0, P5, P13, and P19), or pre-culture immunodepletion of CD4+, CD14+ cells (P0 and P24). Additionally, P0 and P19 virus samples were pre-incubated with soluble recombinant CD4-IgG (sCD4) prior to inoculating PBMCs.

Surprisingly, initial stocks of SIV mac239 _CEMx174_ and SIV 17E-Fr both actually grew better in hPBMCs transiently blocked with Leu3a mAb during initial exposure, than in unblocked controls. Nevertheless, during succeeding passages, CD4 independence was progressively lost (Figure 2A), roughly in parallel with increasing CXCR4 tropism (Figure 2B). Additionally, by passage 24, both SIV strains had lost the ability to infect CD4(−), CD14(−) huPBMCs (see Figure 1B).

### SIV stock strains are not rendered less infectious by pre-incubation with soluble IgG-CD4, and evolve discordant sensitivities during hPBMC passage

As shown in Figure 3, results after pre-incubating SIV with soluble IgG-CD4 were somewhat discordant for the two viruses. For P0 SIV 239 _CEMX174_, sCD4 strongly enhanced infection (as did the CD4 blocking mAbLeu 3a). For P19 SIV 239 _CEMX174_ isolates, which were almost totally blocked by Leu3a, sCD4 suppressed growth by > 80%. Thus, with respect to SIV 239 _CEMX174_, sCD4 pre-incubation reduced infectivity generally comparable to anti-CD4 mAb. However, in the case of SIV 17E Fred, pre-incubation with sCD4 had no effect on CD4 independent infectivity of stock strains or CD4-dependent P19 isolates. Possible reasons for these observed differences are considered in the Discussion section.

### Progressive emergence of variants with specific mutations in the envelope region

We next asked whether there were any common mutations emerging in the strains of SIV that had evolved CD4 dependence (which can be conferred by single mutations [25,26]) and, if so, how quickly they emerged. We sequenced both strands of full length envelope from 15 clones, each, of stock SIV 17E-Fr and stock SIV mac239 _CEMx174_ carried in CEMx174 cells, 15 clones derived from hPBMC passage 7, and 15 clones from passage 24. Initial SIV mac239 _CEMx174_ virus, as well as the neurotropic variant SIV 17E-Fr, contained residues (67 Met and 176 Asn) previously associated with CCR5 tropic, CD4 independent (in macaque cells) SIV recovered from brains of macaques initially infected with WT SIV mac239 [27].

Interestingly, both stock virus strains grown in CEMx174 also retained certain key residues previously associated with CD4 dependent infection in non-human primates, including Met at position 165, Ile at 324, and (in 17E-Fred only) Lys at 573,the CD4 contact point of the conserved C3 loop GGDPE region [16-18]. Thus, in accord with most published sequences of CD4-independent SIV grown in macaque PBMCs, our stock virus sequences suggest stably exposed CKR binding sites as the basis for initial CD4 independence in hPBMCs, rather than complete loss of CD4 affinity.

**Figure 4:**
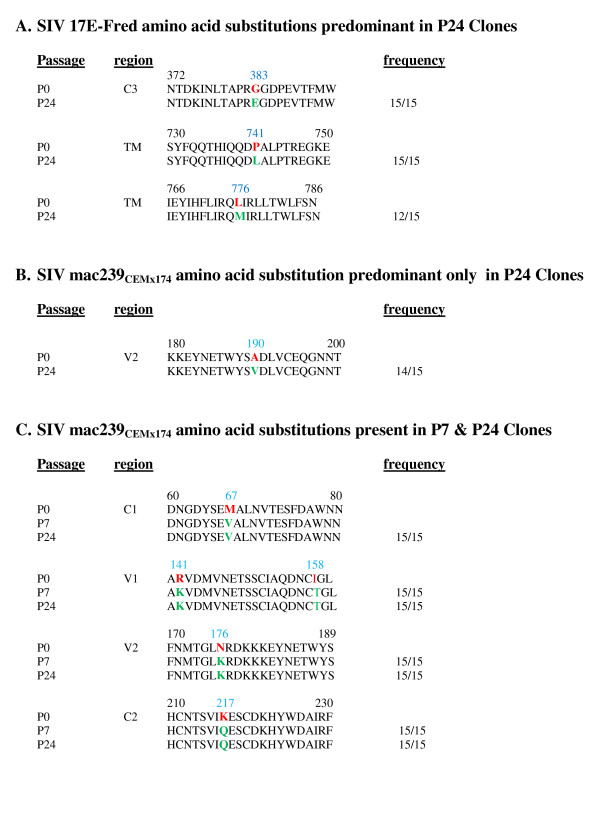
**Predominant changes in amino acids between P0 and P7, or P24.** Single letter sequences are shown for stock (P0), passage 7 (P7) and/or passage 24 (P24) SIV clones, with the position of each change indicated in blue numbers above the P0 sequence, the P0 residue in red, altered residues in green, and the clonal frequency of mutations indicated for passages. The variable (V), conserved (C), or transmembrane (TM) regions in which mutations occur are indicated. SIV 17E-Fred (A) required few mutations to become CD4 dependent. By contrast, SIV 239_CEMx174_required multiple mutations by P24 (B) to become CD4 dependent, with many appearing by P7 and persisting through P24 (C).

Overall, the 15 clones of envelope from SIV 17E-Fr stock virus were > 99.9% homologous at the amino acids (aa) level with eachother and the published sequence of this strain [28]. Despite the observed progressive acquisition of CD4-dependence, stock virus and all subsequently passaged 17E-Fr carried Gly at aa 751, a residue previously shown to contribute to CD4 independence in macaque astrocyte-tropic isolates [15]**.** For SIV mac239 _CEMx174_, all 15 stock clones were, again, remarkably homogeneous, with > 99.9% aa homology among the 15 clones overall, and only two clones having more than a single aa differing from this consensus sequence. Importantly, none of this very limited heterogeneity involved aa positions that subsequently underwent mutation during passage in hPBMCs. Figure 4 aligns regions of the starting nucleotide sequences of the two stock strains (P0) with the predominant mutated sequences, for the respective strains, recovered at P24.

For 17E-Fr, by passage 24, mutations in nucleotide position 1148 (G to A) and 2222 (C to T) were present in all 15 clones sequenced, leading to aa acid changes of Gly to Glu, and Pro to Leu, at aa residues 383 (C3 domain), and 741 (proximal to the transmembrane domain), respectively (Figure 4A). An additional mutation at nucleotide position 2326 (C to A) was seen in 12 of 15 clones from passage 24, yielding a Leu to Met conversion at aa 776. Of these most common mutations, only G to A at nucleotide position 1148 had appeared in 3 of 15 clones by passage 7, suggesting it may have been critical for the step-wise conversion to CD4-dependent phenotype. Glu 813 Arg was present in 8 of 15 passage 24 clones, and several other substitutions were present at frequencies of 4/15 or less.

The pattern of passage acquired mutations for SIV mac239 _CEMx174_, was more complicated than for 17E-Fr, with only a single mutation seen for the first time at passage 24, and not in stock or passage 7 samples (Figure 4B). This was a C to T mutation in 14/15 clones at nucleotide position 569, yielding Ala to Val at aa 190. Unlike the SIV 17E-Fr findings, several additional non-synonymous mutations were found to arise relatively early (during or before passage 7) and persist through passage 24, suggesting these changes also contributed to early partial CD4 dependence and increased fitness in vitro. The most consistent changes, seen in all fifteen of the passage 7, and all fifteen of the passage 24 clones, were: Met 67 Val, Arg 141 Lys, Ile 158 Thr, Asn 176 Lys, Lys 217 Gln, Arg 335 Pro, Arg 341 Lys, 383 Trpinsertion, Tyr 631 His, Asp 766 Gly, Ser 838 Pro. Interestingly, three of these changes were G to A and five were A to G. Notably, almost every mutation emerging during early (P7) hPBMC passage of SIVmac239_CEMx174_ and persisting through P24, resulted in the restoration of an aa present in the canonical SIVmac239 sequence. Additionally, Ile 310 Met, and Tyr 609 Cys mutations were seen in all 15 SIV mac239 _CEMx174_ clones at passage 7 (each reflecting an A to G mutation), but only in 11 of 15 clones at passage 24, with the other clones containing the original stock virus residues. This could have been due either to expansion (i.e., reemergence) of clones carrying the retained stock aa residues, or reverse mutation back to initial sequences (via G to A mutation). As mentioned above, in every case, the emergent dominant mutations present in P7 and P24 clones returned the sequence to the original published SIV mac239 residue, so that P24 SIV mac239 _CEMx174_ was almost identical to the canonical SIV mac239.

## Discussion

An ability to replicate in the setting of severely limited CD4+ targets (e.g., advanced disease or CNS infection) would be a selective advantage for SIV or HIV at various times in the course of a typical infection. CD4 independence might also facilitate xenotransmission to primate species exhibiting dramatically lower binding constants for viral envelope interaction with CD4 [29]. How often this has occurred and to what extent species-specific CD4 has been a barrier to xenotransmission remain unknown. Despite the apparent advantages of CD4 independence, it is the exception rather than the rule, suggesting a strong selective advantage for CD4 dependent viruses.

Reacquisition of CD4-dependence has been observed for neurotropic CD4-independent SIV injected peripherally into monkeys, and has been correlated with, and attributed to, development of neurotropic strain specific neutralizing Abs. Here, we have presented the first evidence that progressive reacquisition of CD4 dependence can evolve in human cell cultures of SIV, over a similar period of time, entirely independent of specific immunity. These observations in no way contradict the importance of immune selection for CD4 dependence in vivo, but do suggest other components, such as cell type specific restriction factors, may exert selective pressure.

The possibility that rare residual CD4+ cells in CD4(−) CD14(−) hPBMCs might be the source of detected initial SIV strain replication is exceedingly remote, as < 1% CD4+ cells scattered among non-infectable neighbors would be unable to support the efficient cell-to-cell transmission that accounts for > 95% of in vitro replication [30]. Also, CD4+ cell contamination cannot explain why viral replication failed to reach detectable levels in identically depleted cultures of late passage, CD4 dependent, SIV.

**Figure 5:**
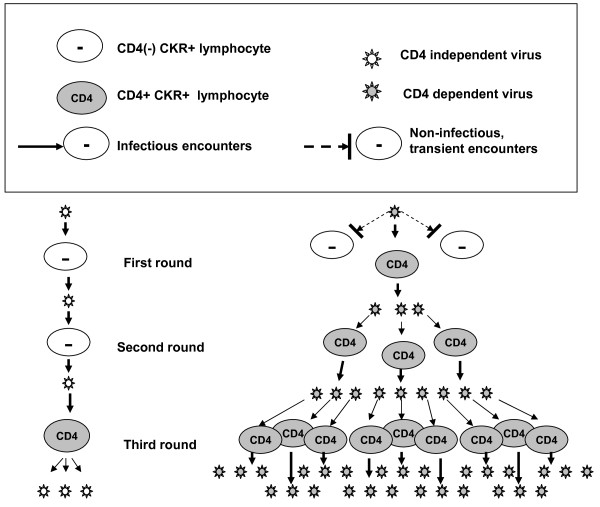
**Modeling the selective advantage of strict CD4 dependence vs. promiscuous entry.** The number of CD4 dependent (shaded) : CD4 independent (unshaded) virions after 3 rounds of replication is 27:3. This illustrative hypothetical example makes the following **s**implifying assumptions: CD4(−) and CD4+ potential host cells in a sufficiently activated state, and bearing the appropriate CKRs, are abundantly present in a ratio of 2 : 1, yielding an encounter with CD4+ targets once in every three virus-cell interactions, on average (transient encounters of CD4 dependent virus with CD4(−) cells are shown only for the first round); the relative level of infectious virus released per infected CD4+ cell is 3x that of an infected CD4(−) cell, and is the same for either strain of virus; the three types of permissive encounters between viruses and host cells have equal efficiencies of entry.

Figure 5 models a 9-fold selective advantage that would be manifest after only three rounds of replication for two hypothetical strains of virus—one CD4 independent and capable of promiscuous entry into any PBMC expressing appropriate surface CKR, the other restricted to CD4+ cells bearing suitable surface CKR. The model assumes identical efficiencies of entry for all permissive combinations of virion and host cell, and makes the further assumptions that CD4(−) and CD4+ lymphocyte targets are available at a ratio of roughly 2: 1, that encounters with targets are stochastically random, and that the reproductive advantage of entering CD4+ vs. CD4(−) cells is just 3-fold. In reality, the reproductive advantage appears to be one or two orders of magnitude greater, and hundreds of replication cycles typically occur prior to detection of seropositivity. Under such conditions, the chance of isolating CD4 independent strains from the peripheral circulation is small.

Up to four-fold increased infection of hPBMCs by stock SIV in the presence of anti-CD4 mAb, seen in several experiments (e.g., Figures 2 and 3), was probably due to more efficient envelope binding of partially capped, actin associated CKRs following Leu3a cross-linking of CD4 [11]. Despite any such enhanced CKR cell surface topography that might facilitate CD4-independent viral infection of transiently Leu3a treated cells, CD4 dependence increased progressively with successive passages, and became nearly absolute by P24.

The reasons for strain differences in the results with soluble Ig-CD4 are not readily apparent, but could relate to one or both of the two established mechanisms for sCD4 mediated inhibition of HIV: steric blocking of cellular CD4 ligation, and induced envelope shedding from virions [31]. In addition, the two strains of initially CD4-independent SIV might show different initial CD4 binding affinities, and different degrees of change in affinities during passage. Indeed, some envelopes have been found not to bind multimeric sCD4 at all, perhaps due to tight trimer packing**,** while certain glycosylation sites have been found to confer relative resistance to sCD4 inhibition [32]. Moreover, effects of sCD4 can include concentration dependent enhancement of infectivity, which may vary with the presence or absence of surface CD4 expression of target cells [31,33-35]**.** Mono- and multi- meric sCD4 were previously shown to enhance SIV mac239 infectivity on CD4+ and CD4(−) CCR5+ cells [31,33-35]**,** presumably by stabilizing CKR binding sites that are already partially exposed, and we suspect that this mechanism is operative for our stock SIV mac239 _CEMx174_ as well. By passage 19, this enhancement was replaced by predominant inhibition. The Leu3a blocking data support the interpretation that exposure of cryptic CKR binding sites in late passage isolates requires prior engagement of CD4. In this context, either shedding of envelope, or steric interference with envelope-CKR docking, could account for the suppressive effect of sCD4.

In contrast, the relative lack of effect of sCD4 co-incubations on either initial or late passage SIV 17E-Fred suggests that changes in CD4 binding affinity were not involved in the acquisition of CD4 dependence. Rather, new conformational masking of CKR binding sites may have evolved in the late-passage isolates, requiring relatively prolonged contact with surface CD4 to allow fusogenic binding. This observation is consistent with the minimal binding of envelope to soluble CD4, as has been previously described for some isolates, or binding with only subtle changes in SIV env conformation, as seen in recent cryo-electron studies [36]. Thus, mechanistic interpretation of discordant sCD4 effects is not straightforward, but these experiments underscore the fact that the structural/functional differences accumulating between initial and late passage SIV mac239 _CEMx74_ viruses were somewhat different in kind from those accumulating in SIV-17E-Fred.

The observed switch from mixed CCR5 and CXCR4 usage in hPBMCs to predominantly CXCR4 usage by both strains has been described for SIV cultured in hPBMCs [3]**,** and may be functionally related to acquisition of CD4-dependence [25]**.** Lauren et al. [37] have suggested the two are interrelated properties for SIVsm, but this is not an absolute requirement. Which of the envelope mutations associated in our experiments with CD4 dependence determine CKR tropism is undefined. The exact changes at aa positions 47 in the C1 domain, and aa 316, 324, and 328 in the V3 loop, described by Del Prete et al. [38] as conferring CXCR4 dependence on SIV mac239 were not seen. However, mutations did occur at nearby positions within the same regions (aa positions 67, 334, 340), and may have played a similar role in the context of the other mutations arising in SIVmac239 _CEMx174_ as it developed increasing CD4 dependence.

PHA/IL-2 stimulated PBMCs contain more CXCR4+ than CCR5+ expressing cells [39], and this difference is more pronounced in CD4(+) vs. CD4(−) populations in culture [Table 1, but relative abundance of CXCR4 vs. CCR5 cannot be the sole reason for the tropism switch, as both R5 tropic stock strains were grown in the CEMx174 line that express ~ 20,000 CXCR4 surface receptors per cell and no detectable surface CCR5. In vivo switching of CCR5 tropic to CXCR4 tropic SHIV has also recently been described in macaques, and is correlated with enhanced, rather than de novo, binding to CD4, based on a more open molecular conformation that may facilitate heterotypic binding to CKRs [40].

The early appearance of G to A mutations in both SIV mac239_CEMx174_ and 17E-Fr, along with the apparent back and forth mutations between G and A seen with SIV mac239 _CEMx174_ in the course of evolving binding requirements, suggests a role for APOBEC 3 mediated G to A mutations in rapidly generating optimal viral fitness for growth in hPBMCs. This could help account for why primate retroviruses have evolved only partial inhibition of APOBEC 3 activity. It is interesting to note that, in all but one instance, mutations acquired by SIVmac239_CEMx174_ restored the sequence to that of the canonical SIV mac239.

## Conclusions

Our findings suggest that in the absence of HIV specific immunity, there are still strong selective pressures favoring replication of SIV clones that limit their entry to CD4+ cells. Seen in this light, surface CD4 becomes an important “Welcome” sign for primate retroviruses, signaling the most supportive host cells, rather than merely an opportune foothold by which to gain entry. The unique intracellular features of CD4+ T lymphocytes, compared with minimally productive CD8+ T cells, NK cells, or B lymphocytes, accounting for such strong selective pressure in vitro and in vivo remain unknown, but may hold the key to future clinical interventions. Recent studies have identified restriction factors for DCs [41,42], and it is possible that these or other intracellular factors limiting primate lentivirus replication will be found in CD4(−) lymphocytes, as well.

## Methods

### Cells and reagents

The human CEMx174 cell line was originally obtained from ATCC (Manassas, VA). Human PBMCs were stimulated for 3 days with 5μg/ml of phytohemagglutinin (PHA) and 2 U (IL-2) in 10% FCS RPMI 1640. CD4+ or CD8+ cells were enriched by negative depletion using, respectively, anti-CD8 or anti-CD4 magnetic beads, in combination with anti-CD14 magnetic beads (Dynal, Lake Success, N.Y.) at saturating concentrations. The purity of T-cell subsets was determined for each subset by flow cytometric analysis of cells immunostained with 20 μg/ml anti-CD4 and anti-CD8 monoclonal antibodies (MAbs) detected with FITC-goat anti-mouse IgG. HIV blocking anti-CD4 MAb Leu3A (BD Biosciences, San Jose, CA, USA) was used at 10 μg/ml. Soluble CD4-IgG (Progenics) was used at 10 μg/ml during 30 minute pre-incubations with SIV, just prior to infection of cells.

Flow measurement of CKR expression on normal hPBMCs stimulated with PHA + IL-2 employed anti-CCR5 mAb 2D7 for flow cytometricstainining developed with goat anti-mouse IgG-FITC; anti-CXCR4 mAb 12 G5 was detected by flow cytometetry with goat anti-mouse IgG-PE, In these experiments, anti-CD4 mAb was conjugated to either FITC or PE, depending on the CKR mAb being used.

This work was approved by the Institutional Review Board of the Bloomberg School of Public Health, in conformance with the Helsinki Declaration on rights of human subjects.

### Virus infection

SIVmac239 and SIV17E-Fr [28], a kind gift from Dr. Janice Clements, were passaged in human derived CEMx174 cells and purified from culture supernatants by sucrose density gradient centrifugation. The resulting SIV17E-Fr was essentially identical to the starting strain, but the SIV mac239 derived stock virus was found to differ in 15 of 15 clones from the published sequence of SIVmac239 by three mutations previously associated with CD4 independence. Thus, we refer to this CEMx174 stock strain throughout the text as SIV mac239_CEMx174._

CEMx174 passaged stock viruses were serially passaged in PHA + IL-2 (2 IU) stimulated human PBMCs obtained from normal control donors. Cultures were refed with fresh medium twice weekly, and passaged into fresh allogeneic human PBMCs every week. PHA + IL-2 stimulated PBMCs were pretreated with Leu3A (or mock treated) for 30 minutes on ice, and infected with stock SIV (10^3^ TCID_50_), or SIV from various passages (culture supernatant containing ~ 5 ng/ml p27) for 2 hrs at 37 °C. After infection, the cells were washed free of virus x 2, and plated at 1x10^6^ per well in quadruplicate wells of a 48-well culture plate. Supernatants collected on days 0, 3, 7 and 10 were assayed for p27 by enzyme immunoassay (EIA, Coulter Immunology). Day 7 and/or day 10 p27 values were used to assess relative rates of growth among control and experimental groups. Groups were statistically compared by two-way Student’s T- test.

### PCR amplification and sequencing of SIV env sequences from cell culture supernatants

Genomic DNA was extracted from culture supernatants of SIVmac239 _CEMx174_ and SIV 17E-Fr infected human hPBMCs with QIA Amp DNA Blood mini Kit (Qiagen), according to the manufacturer’s recommendations. Genomic DNA was used for PCR amplification of the entire SIV *env* gene in a hot start PCR (Taq Master Mix Kit, Qiagen) according to manufacturer’s instructions in a standard PCR buffer, and primers at a concentration of 0.5 uM, P1 (5′ GAGCAATCACGAAAGAGAAGAAGAAC-3′) and P2 (5′ CTGTC CCTGAT TGTATTTCTGTCCC-3′) for the first round and primers P3 (5′ GCTCTAGAATGGGATG TCTTGGGAATCAGCTGC-3′) and P4 (5′ CGGATCCTCACAAGA GAGTGGCTCAAGCCC-3′) for the second round, as previously described [43].

PCR products were run on a 0.7% low-melt agarose gel, and the 2.6 kb env product was extracted and cloned into the pCR2.1-TOPO vector (TOPO TA cloning kit, Invitrogen) according to manufacturer’s instructions. Fifteen full-length *env* clones from each stock SIV virus, passages 7 and 24 were randomly selected and sequenced bi-directionally at the Johns Hopkins Sequencing Facility using the M13F and M13R primers with the dye terminator cycle sequencing method and 3730 DNA Analyzer (Applied Biosystems). Sequence analysis was done using SeqMan II, part of Lasergene v 4.03 (DNA Star Inc.). Multiple sequence alignment was done using CLUSTAL X with weighted residue weight table [44], built into the MegAlign, part of Lasergene version 4.03 (DNA Star Inc.).

## Competing interests

The authors declare that they have no competing interests.

## Authors’ contributions

DS conceived the project, participated in the design and interpretation of the experiments, and drafted the manuscript. SI designed experiments, analyzed the genetic data, and participated in drafting and editing the manuscript. Both authors approved the final manuscript.
